# Mutation Analysis of *IDH1* in Paired Gliomas Revealed *IDH1* Mutation Was Not Associated with Malignant Progression but Predicted Longer Survival

**DOI:** 10.1371/journal.pone.0067421

**Published:** 2013-06-28

**Authors:** Yu Yao, Aden Ka-Yin Chan, Zhi Yong Qin, Ling Chao Chen, Xin Zhang, Jesse Chung-Sean Pang, Hiu Ming Li, Yin Wang, Ying Mao, Ho-Keung NG, Liang Fu Zhou

**Affiliations:** 1 Department of Neurosurgery, Huashan Hospital, Fudan University, Shanghai, China; 2 Department of Anatomical and Cellular Pathology, The Chinese University of Hong Kong, Hong Kong; 3 Shenzhen Research Institute, The Chinese University of Hong Kong, Shenzhen, China; 4 Department of Neuropathology, Huashan Hospital, Fudan University, Shanghai, China; NIH/NCI, United States of America

## Abstract

Recurrence and progression to higher grade lesions are characteristic behaviorsof gliomas. Though IDH1 mutation frequently occurs and is considered as an early event in gliomagenesis, little is known about its role in the recurrence and progression of gliomas. We therefore analysed *IDH1* and *IDH2* statusat codon 132 of *IDH1* and codon 172 of *IDH2* by direct sequencing and anti-IDH1-R132H immunohistochemistry in 53 paired samples and their recurrences, including 29 low- grade gliomas, 16 anaplastic gliomas and 8 Glioblastomas. *IDH1/IDH2* mutation was detected in 32 primarytumors, with 25 low- grade gliomas and 6 anaplastic gliomas harboring *IDH1* mutation and 1 low- grade glioma harboring *IDH2* mutation. All of the paired tumors showed consistent *IDH1* and *IDH2* status. Patients were analyzed according to *IDH1* status and tumor-related factors. Malignant progression at recurrence was noted in 22 gliomas and was not associated with *IDH1* mutation. Survival analysis revealed patients with *IDH1* mutated gliomas had a significantly longer progression-free survival (PFS) and overall survival (OS). In conclusion, this study demonstrated a strong tendency of *IDH1/IDH2* status being consistent during progression of glioma. *IDH1* mutation was not a predictive marker for malignant progression and it was a potential prognostic marker for gliomas of Chinese patients.

## Introduction

Gliomas are the most common primary brain tumors, accounting for 80% of malignant central nervous system neoplasms [1].Recent genome-wide mutational analysis has demonstrated that the incidence of *IDH1* mutations in gliomas ranges from 5% in primary glioblastoma (GBM) to 70% in anaplastic astrocytomas (AA) and 80% in secondary GBM [2]–[6]. Patients with high-grade astrocytomas with *IDH1* mutations were reported to have a better survival [6].

The *IDH1* gene is located on 2q33.3 and its mutation has been described in a very restricted number of human cancers including gliomas [3], [7], [8].The most common *IDH1* mutation is a heterozygous missense mutation with a change of guanine to adenine at position 395 (G395A), leading to the replacement of arginine by histidine at codon132 (IDH1-R132H) at the enzymatic active site [10]. *IDH1* mutation has been shown to occur in early stage of gliomagenesis [5]. The pathogenesis of IDH1-R132H–related tumorigenesis is rapidly being elucidated. Not only does loss of function occur with reduced production of α-ketoglutarate (α-KG) from isocitrate, the R132H mutation also confers a gain of function to the mutant IDH1, which converts α-KG to 2-hydroxyglutarate (2-HG) [11]. Accumulation of this oncometabolite induce sextensive DNA hypermethylation, leading to genome-wide epigenetic changes and predisposing cells toward neoplastic transformation [12].

In spite of all the studies, the role of *IDH1* mutation in the recurrence of gliomas is unknown. There have been few studies in which paired gliomas at primary presentation and recurrence were studied by molecular means. In the present study, we investigated the mutation status of *IDH1* and *IDH2* in 53pairs of primary and recurrent gliomas. All pairs showed consistent *IDH1/IDH2*status.Correlation analysis with clinicopathological parameters revealed that*IDH1* mutation was not associated with malignant progression but was a potential prognostic marker for progression-free survival (PFS) and overall survival (OS) in astrocytomas.

## Patients and Methods

### Ethics Statement

This study was approved by the Ethics Committee of Shanghai Huashan Hospital and the New Territories East Cluster-Chinese University of Hong Kong Ethics Committee.

### Patients and Tissue Samples

Records of patients with glioma diagnosed in the Department of Neurosurgery, Huashan hospital (Shanghai, China) and Department of Anatomical and Cellular Pathology, Prince of Wales Hospital (Hong Kong) between 1990 and 2011 were reviewed. 53paired cases were retrieved where formalin-fixed paraffin embedded (FFPE) tissues were available from primary presentations and recurrences (Table S1). Haematoxylin &eosin (H&E) stained sections of each tumor were reviewed and graded according to the 2007 WHO classification of tumors of central nervous system.

### Mutation Analysis of IDH1/IDH2

Mutational hotspots of *IDH1*at codon 132 and *IDH2* at codon 172 were evaluated by direct sequencing. Representative tumor area scrapped off from dewaxed sections into microfuge tubes were resuspended in 10 mMTris-HCl buffer, pH 8.5. Proteinase K was added to a final concentration of 2g/l and the mixture was incubated at 55°C for 2 hours and then at 98°C for 10 min. The PCR mixture of 10 µl volume contained 1–2 µl of crude cell lysate, 10 mMTris-HCl (pH 8.3), 50 mMKCl, 2.5 mM MgCl_2_, 0.2 mM of each deoxyribonucleoside triphosphate, 0.4 mM of each primer (IDH1-F: 5′-CGGTCTTCAGAGAAGCCATT-3′ and IDH1-R: 5′-CACATTATTGCCAACATGAC-3′; IDH2-F: 5′-AGCCCATCATCTGCAAAAAC-3′ and IDH2-R: 5′-CTAGGCGAGGAGCTCCAGT-3′) [5], [9] and 0.2 units of AmpliTaq Gold DNA polymerase (Applied Biosystems, Hong Kong). PCR was initiated at 95°C for 10 min, followed by 45 cycles of 95°C for 20 sec, 60°C for 20 sec and 72°C for 30 sec, and a final extension step of 72°C for 3 min. Products were then treated with exonuclease I and alkaline phosphatase (TakaRa, Japan). Sequencing was performed using BigDye Terminator Cycle Sequencing kit v1.1. The products were resolved in the Genetic Analyzer 3130xl and analyzed by Sequencing Analysis software. All base changes were confirmed by sequencing of a newly amplified fragment.

### Immunohistochemistry of IDH1-R132H

FFPE tissue sections of 4 micron thickness were deparaffinized in xylene and rehydrated in graded alcohols. Antigen retrieval was carried out by treating the sections in 1 m Methylenediaminete traacetic acidsolution (pH 8.0) in a microwave oven. After antigen retrieval, the slides were processed by BenchMark XT automated tissue staining systems (Ventana Medical Systems, Inc., Tucson, U.S.A.) using validated protocols. Tissue sections were incubated at 37°C for 32 min with mouse monoclonal anti-IDH1-R132H antibody (1∶50 dilution; Dianova, Hamburg, Germany) followed by incubation with UltraView HRP-conjugated multimer antibody reagent (Ventana). Antigen detection was performed using Ultra View diamino benzidine chromogen step (Ventana). Tissues were counterstained with hematoxylin. The presence of cytoplasmic staining indicated positivity for IDH1-R132H.

### Statistical Analysis

Statistical analysis was performed by PASW Statistics 18 (version 18.0.0; SPSS, Inc.). The Chi square test (or Fisher exact test when one subgroup was ≤5) was used to examine association between categorical data. Overall survival (OS) was defined as the time between the diagnosis and death or last follow-up. Progression-free survival (PFS) was defined as the time between the diagnosis and first unequivocal clinical or radiological sign of progressive diseases. Survival curves were plotted by Kaplan-Meier method and analyzed by Log-rank test. Multivariate analysis for independent prognostic marker was performed by Cox-proportional hazards model. Two-sided p-value less than 0.05 was considered as statistically significant.

## Results

### Primary Tumors

The primary tumor cohort consisted of 29 low grade gliomas (WHO grade II) (17 diffuse astrocytomas, 7 oligoastrocytomas and 5 oligodendrogliomas), 16 anaplastic gliomas (WHO grade III) (8 anaplastic astrocytomas, 3 anaplastic oligodendrogliomas, 1 anaplastic oligoastrocytoma, 1 anaplastic ganglioglioma and 3 anaplastic ependymomas) and 8 glioblastomas (GBM) (WHO grade IV). WHO grade II was defined as low grade glioma (LGG), while WHO grade III and IV were defined as high grade.The mean and median age of the patients was39.5and 38 years, respectively (range 5 to 67). The male/female ratio of the cohort was 1∶0.83. 96% (51/53) of cases were supratentorial tumors and 4% (2/53) of cases were infratentorial tumors.

### Recurrent Tumors

#### Recurrent tumors with malignant progression

Malignant progression occurred in 42% (22/53) of the primary tumors. 55% (16/29) of LGGs underwent malignant transformation upon recurrence, with eight cases recurred as anaplastic gliomas while eight cases progressed to GBM. Similarly, 38% (6/16) of anaplastic gliomas progressed to GBM upon recurrence. (Table 1).

**Table 1:** Histological grading of 53 pairs of primary and recurrent gliomas.

		Recurrent tumor			No. of cases recurred as same histological grade	No. of cases recurred with malignant transformation	Total no. of tumors
		LGG	AG	GBM			
Primary tumor	LGG	13	8	8	13 (45%)	16 (55%)	29
	AG	0	10	6	10 (63%)	6 (38%)	16
	GBM	0	0	8	8 (100%)	0 (0%)	8
	Total	13	18	22	31 (59%)	22 (42%)	53

LGG: low grade glioma; AG: anaplastic glioma; GBM: Glioblastoma.

#### Recurrent tumors with histological grade same as primary tumor

58% (31/53) of the primary tumors had recurrence with same histological grade as the corresponding primary tumors, including 45% (13/29) of LGGs, 63% (10/16) of anaplastic gliomas and 100% (8/8) of GBM. (Table1).

### IDH1/IDH2 Mutation

*IDH1/IDH2* mutation analysis by direct sequencing and anti-IDH1-R132H immunohistochemistry revealed 60% (32/53) of the primary tumors harboring*IDH1* or *IDH2* mutations, which included90% (26/29) of LGG, 38% (6/16) of anaplastic gliomas and none of the primary GBM. All of the recurrent tumors showed consistent *IDH1/IDH2* status as the corresponding primary tumors. Result of anti-IDH1-R132H immunohistochemistry was 100% concordant with direct sequencing. Among the 32 mutations detected, 91% (29/32) was IDH1-R132H, 3% (1/32) was IDH1-R132S, 3% (1/32) IDH1-R132G and 3% (1/32) was IDH2-R172K. *IDH1/IDH2* mutation was observed in 90% (26/29) of primary LGGs, 38% (6/16) of primary anaplastic gliomas and none (0/8) of the primary GBM. Similarly among the recurrent tumors, *IDH1/IDH2* mutation was detected in 77% (10/13) of LGGs, 61% (11/18) of anaplastic gliomas and 50% (11/22) of GBM, with 79% (11/14) of secondary GBM harbored the mutation. (Table 2).

**Table 2:** *IDH1/IDH2* status of primary and recurrent gliomas.

	Initial tumor			Recurrent tumor		
	*IDH1/IDH2* mutant	*IDH1/IDH2* wild type	Total no.	*IDH1/IDH2* mutant	*IDH1/IDH2* wild type	Total no.
LGG	26 (90%)	3 (10%)	29	10 (77%)	3 (23%)	13
AG	6 (38%)	10 (63%)	16	11 (61%)	7 (39%)	18
GBM	0 (0%)	8 (100%)	8	11 (50%)	11 (50%)	22

LGG: low grade glioma; AG: anaplastic glioma; GBM: Glioblastoma.

Only one case of oligoastrocytoma (WHO grade II) harbored *IDH2* mutation and progressed to anaplastic oligoastrocytoma upon recurrence.

### Relationshipbetween IDH1 Mutation and Malignant Transformation

In LGGs, 94% (15/16) of tumors with malignant transformation upon recurrence harbored *IDH1* mutation, whereas 77% (10/13) of tumors recurring without malignant transformationharbored *IDH1* mutation(p = 0.299).One case of oligoastrocytoma (WHO grade II) harbored *IDH2* mutation and recurred as anaplastic oligoastrocytoma (WHO grade III). In patients with anaplastic gliomas, 50% (3/6) of tumors progressing to GBM upon recurrence were *IDH1* mutated and 30% (3/10) of tumors without malignant transformation upon recurrence had *IDH1* mutation (p = 0.607). Therefore, we did not observe any association between *IDH1* mutation and malignant transformation(Table 3).

**Table 3:** Correlation between *IDH1*mutation and malignant transformation in gliomas.

Tumor grade	*IDH1* mutant	*IDH1* wild type	p-value
LGG →LGG	10 (77%)	3 (23%)	0.299
LGG → AG/GBM	15 (94%)	1 (6%)	
AG → AG	3 (30%)	7 (70%)	0.607
AG → GBM	3 (50%)	3 (50%)	
GBM → GBM	0	8	N/A

LGG: low grade glioma; AG: anaplastic glioma; GBM: Glioblastoma.

### Survival analysis

**Figure 1:**
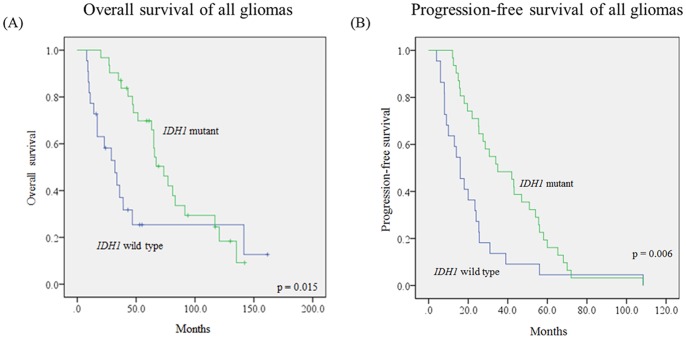
Kaplan-Meier survival curves comparing OS (A) and PFS (B) in all gliomas with or without IDH1 mutation. (A) Median OS was 73.5 months for *IDH1* mutated gliomas and 32 months for *IDH1* wild type gliomas (p = 0.015, Log-rank test). (B) Median PFS was 34.9 months for *IDH1* mutated gliomas and 16 months for *IDH1* wild type gliomas (p = 0.006, Log-rank test).

**Figure 2:**
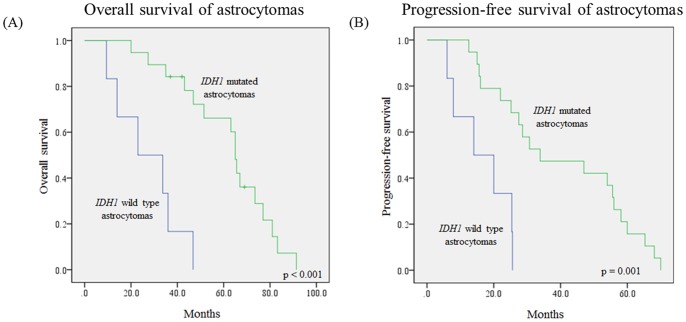
Kaplan-Meier survival curves comparing OS (A) and PFS (B) in astrocytomas (AII and AAIII) with or without IDH1 mutation. (A) Median OS was 65 months for *IDH1* mutated astrocytomas and 23 months for *IDH1* wild type astrocytomas (p<0.001, Log-rank test). (B) Median PFS was 33.9 months for *IDH1* mutated astrocytomas and 14 months for *IDH1* wild type astrocytomas (p = 0.001, Log-rank test).

Survival data was available in all of the patients in this study. The median follow-up time, PFS and OS were 161.6 months, 25.4 months and 63.1 months, respectively. Univariate analysis showed advanced WHO grade, age over 50 years, astrocytic phenotype and wild type *IDH1* were poor prognostic factors for OS (Figure S1a to 1c, Figure 1A, Table 4). Advanced WHO grade, age over 50 years and wild type *IDH1* were associated with shorter PFS (Figure S1d to 1f, Figure 1B, Table 4). Further analysis in astrocytomas (AII and AAIII) revealed the association between *IDH1* mutation and prognostic outcome. Patients with *IDH1* wild-type astrocytomas had shorter OS (median 65 months) and PFS (median 33.9 months) than those with *IDH1*mutated astrocytomas (median OS 23 months, p<0.001; median PFS 14 months, p = 0.001) (Figure 2A and 2B).

**Table 4:** Univariate analysis of overall survival (OS) and progression-free survival (PFS).

Variable		Median OS (months)	p-value	Median PFS (months)	p-value
Age	below 50	65	0.014	27.5	<0.001
	50 or above	20		12.1	
WHO grade	Grade II	73.5	<0.001	34.9	<0.001
	Grade III	37.2		19.6	
	Grade IV	11		8	
Histological phenotype	Astrocytic	46.8	<0.001	25.2	0.277
	Oligodendroglial	135.2		25.4	
*IDH1* status	*IDH1* wild type	32	0.015	16	0.006
	*IDH1* mutant	73.5		34.9	

Multivariate analysis by Cox-proportional hazards model identified age (p = 0.01), WHO grade (p = 0.001), tumor phenotype (p<0.001) and *IDH1*status (p = 0.002) as independent prognostic factors in OS in our cohort of gliomas. Age (p = 0.03) and *IDH1* status (p = 0.006) were shown to be independent prognostic factors in PFS. (table5).

**Table 5:** Multivariate analysis of overall survival (OS) and progression-free survival (PFS) by Cox proportional hazards model.

		OS			PFS		
		HR	95% CI	p-value	HR	95% CI	p-value
Age		1.04	1.01 to 1.07	0.01	1.03	1.00 to 1.05	0.03
WHO grade	Grade II	0.08	0.02 to 0.33	0.001	0.51	0.17 to 1.57	0.24
	Grade III	0.23	0.06 to 0.98	0.046	1.03	0.33 to 3.17	0.96
	Grade IV	1	n/a	0.001	1	n/a	0.17
Histological phenotype	Astrocytic	6.98	2.37 to 20.58	<0.001	1.14	0.57 to 2.30	0.72
	Oligodendroglial	1	n/a		1	n/a	
*IDH1*status	*IDH1* wild type	4.74	1.73 to 12.98	0.002	3.60	1.45 to 8.95	0.006
	*IDH1*mutant	1	n/a		1	n/a	

## Discussion

Watanabe et al. dissected multiple biopsies from the same patients and found that *IDH1*mutations always preceded acquisition of*TP53*mutation or loss of 1p/19q [5].This genetic evidence suggests that *IDH1*mutations are early genetic events in the development of glioma from a cell-of-origin that can give rise to both astrocytes and oligodendrocytes. To date, little is known about the role of *IDH1* and its clinical implications in the processes of glioma progression, particularly in Chinese patients. Previous reports were mainly focused on analysis of *IDH1* status in primary gliomas or secondary gliomas. Thus, the significance of *IDH1*in paired gliomas, especially its role in predicting malignant progression, remains to be further defined. In our study,we investigated the *IDH1* and *IDH2* status of 53 pairs of primary and recurrent gliomas by direct sequencing and anti-IDH1-R132H immunohistochemistry. All of the primary gliomas showed consistent *IDH1/IDH2* status as the corresponding recurrent gliomas, includingthe three cases of rare mutant (IDH1-R132S, IDH1-R132G and IDH2-R172K). No association was observed between *IDH1* mutation and malignant transformation. Together with the fact that *IDH1* mutation is an early event in gliomagenesis, its constant statusthroughout the tumor evolution and absenceof association with malignant transformation suggest that *IDH1* mutation is likely involved in tumor initiation instead of malignant progression [22]. Interestingly, a very recent paper by Lass et al. [22] showed that a small number of gliomas changed its *IDH1* status in recurrence.

Evidence has accumulated in the literature regarding the prognostic impact of *IDH1* mutation in gliomas, particularly high grade gliomas [4], [6], [21], [23]–[26]. The prognostic significance of *IDH1* mutation in LGG is more debatable. Dubbink et al. investigated the *IDH1/IDH2* status in 49 low grade astrocytomas and demonstrated the association between *IDH1* mutation and improved OS [27]. In another study, Houllier et al. analysed the clinical and molecular data of 271 LGGs and identified *IDH1/IDH2* mutation as an independent prognostic marker in OS of LGG after adjusting for age, gender, Karnofsky performance status (KPS), histology, type of surgery, chromosome 1p/19q status and MGMT methylation [18]. By studying 404 gliomas (including 100 LGGs), Sanson et al. also showed the independent prognostic significance of *IDH1* mutation in OS of gliomas by multivariate analysis adjusting for age, histological grade, type of surgery, postoperative treatment and molecular alterations (including 1p/19q codeletion, MGMT methylation and EGFR amplification) [23].In a study investigating various molecular markers (including *TP53* mutation, *MGMT* promoter methylation, 1p/19q codeletion and *IDH1* mutation) of 139 LGGs, Hartmann et al. found that *IDH1* mutation was the strongest prognostic marker for OS regardless of histology [31].On the other hand, *IDH1/IDH2* mutation was of no prognostic value in a study by Kim et al.investigating*IDH1/IDH2* mutation, 1p/19q codeletion and *TP53* mutation in 360 LGGs [28]. Ahmadi et al. also evaluated 100 diffuse astrocytomas and found the lack of association between *IDH1* mutation and clinical outcome in terms of OS, PFS and time to malignant progression [29]. Differences in methodology perhaps partially explained such discrepancy in their conclusions regarding prognostic impact of *IDH1* mutation. OS was calculated from the date of first symptom in the study by Ahmadi et al. but most other studies, including ours, calculated OS from the date of histological diagnosis or date of first surgery. Secondly, patients in Ahmadi’s study were treated with nitrosourea-based chemotherapy as adjuvant treatment but in Hartmann’s study which demonstrated survival benefit of *IDH1* mutated LGG, adjuvant treatment was alkylating agents.Additionally, in contrast to most studies about *IDH1/IDH2* mutation in gliomas in the literature which investigated primary samples, we studied paired primary and recurrent gliomas. Such differences in methodology potentially influenced the evaluation of prognostic impact of *IDH1* mutation in LGG. In our study, *IDH1* mutation was associated with longer OS and PFS in 53 patients suffering from various grades of glioma, particularly in astrocytic tumors. Due to the relatively small size of our cohort and only 3 LGGs (2 adult AII, 1 paediatric OAII) were *IDH1* wild type, statistical analysis of *IDH1* mutation in LGG in our cohort was not performed. Further study with larger cohort would be needed to address the prognostic value of *IDH1* mutation in LGG of Chinese patients. Nevertheless, our study has provided further evidence for the prognostic impact of *IDH1* mutation in gliomas in general in Chinese patients.

In contrast to chromosome 1p/19q codeletion requiring fluorescence in-situ hybridization (FISH)analysis and MGMT promoter methylation requiring methylation-specific PCR (MSP), which are important diagnostic and predictive markers of glioma, *IDH1* status could be readily evaluated by anti-IDH1-R132H immunohistochemistry for the most common mutant or by PCR followed by direct sequencing for all the mutant of the two mutation hotspots of *IDH1* and *IDH2*.Our study examined the *IDH1/IDH2* status of 53 pairs of primary and recurrent gliomas. The concordance rate of the two assays was 100%, confirming the reliability of mutation analysis in our study.

Malignant progressionat recurrence is a crucial phenomenonin patients suffering from gliomas. We have previously evaluated various molecular alterations in a series of microdissected primary GBM and paired astrocytic tumors and revealed that low grade areas and high grade areas of primary GBM had more similar genetic abnormalities comparing with paired low and high-grade tumors underwent malignant progression, suggesting that additional molecular aberrations accumulate during malignant transformation [32]. Authors of several recent studies have examined the histological grade and molecular alterations in order to identify biomarkers for predicting malignant progression. In a study of 33 WHO grade II astrocytomas by Yue et al. [15], expression of Ki-67 was significantly associated with malignant progression, suggesting that tumors expressing higher Ki-67 may have an inherently faster growth rate and thus recur faster in the setting of gross-total or subtotal resection. Ishii et al. reported that the presence of *TP53* mutation in WHO Grade II astrocytoma was associated with malignant progression and shorter PFS, whereas tumors without *TP53* mutation recurred and progressed to malignancy without the change in *TP53* status [16]. In this study, we evaluated the relationship between progression of glioma and *IDH1* status but no association between *IDH1* status and malignant progression was observed.

Though many studies demonstrated that *IDH1* mutation was an important biomarker in glioma, mechanism of *IDH1* mutation in glioma was not yet fully determined. Zhao et al. demonstrated the accumulation of hypoxia-inducible factor subunit (HIF-1α) due to reduced formation of α-KG in *IDH1*-mutated glioma cells, suggesting that activation of the HIF-1 pathway may be one of the oncogenic mechanisms of *IDH1* mutation [10].Dang et al. [11] further discovered the neomorphic gain of function of the IDH1-R132H mutant protein in converting α-KG to α-HG, an oncometabolite inhibiting multiple α-KG-dependent dioxygenases and leading to genome-wide histone and DNA methylation alterations [12].Turcan et al. [13]unmasked the in vivo effect of *IDH1* mutation in primary human astrocytes by showing the IDH1-R132H mutation induced histone alterations and extensive DNA hypermethylation, which actually remodel the methylome and establish the glioma CpG island methylator phenotype (G-CIMP), a subset of glioma with distinct genomic and clinical characteristics [30].

In summary, our study is the first study in investigating the *IDH1/IDH2* status in paired primary and recurrent gliomas in Chinese patients. We have shown consistent *IDH1/IDH2* status in the progression of gliomas and lack of association between *IDH1*mutation and malignant progression. Patients with *IDH1* mutated gliomas had longer OS and PFS, suggesting *IDH1* mutation as a potential prognostic marker in gliomas for Chinese patients.

## Supporting Information

Figure S1 **Kaplan-Meier survival curves comparing OS and PFS in gliomas with advanced WHO grade, age and astrocytic phenotype.** (a–c) Comparison of Kaplan–Meier OS curves according to advanced WHO grade, age over 50 years and astrocytic phenotype. (d–f) Comparison of Kaplan–Meier PFS curves according to advanced WHO grade, age over 50 years and astrocytic phenotype.
(TIF)

Table S1 **Clinical data and IDH status of 53 patients with paired primary and recurrent gliomas.**
(DOCX)
