# Supplementary figures and images for: Mutation Analysis of IDH1 in Paired Gliomas Revealed IDH1 Mutation Was Not Associated with Malignant Progression but Predicted Longer Survival
Source: PLoS One. 2013 Jun 28;8(6):e67421. doi: 10.1371/journal.pone.0067421 (PMC3696098; doi:10.1371/journal.pone.0067421)

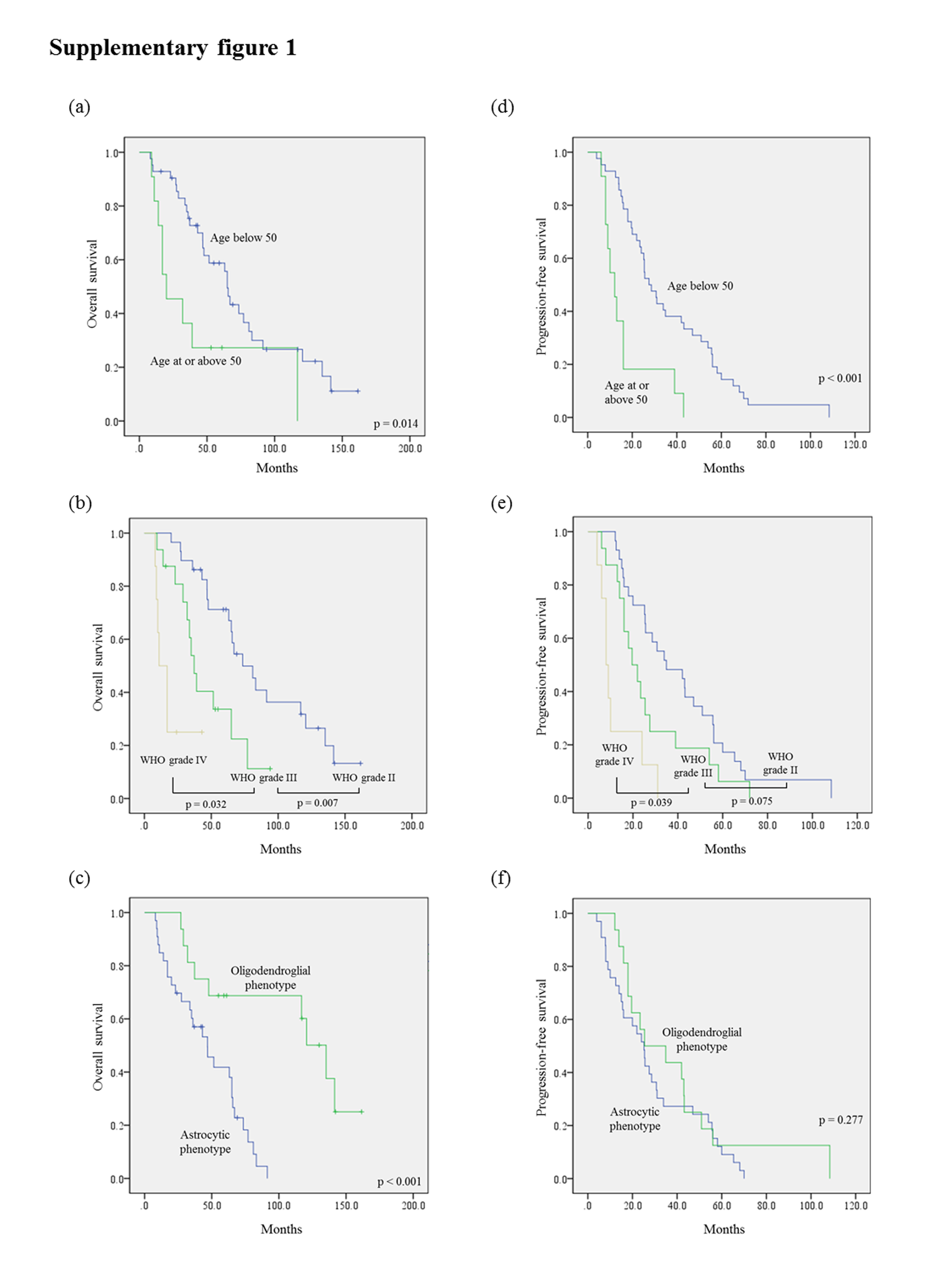

Supplement: Figure S1 — Kaplan-Meier survival curves comparing OS and PFS in gliomas with advanced WHO grade, age and astrocytic phenotype. (a–c) Comparison of Kaplan–Meier OS curves according to advanced WHO grade, age over 50 years and astrocytic phenotype. (d–f) Comparison of Kaplan–Meier PFS curves according to advanced WHO grade, age over 50 years and astrocytic phenotype. (TIF) [file pone.0067421.s001.tif]
