# Supplementary material for: Mutation Analysis of IDH1 in Paired Gliomas Revealed IDH1 Mutation Was Not Associated with Malignant Progression but Predicted Longer Survival
Source: PLoS One. 2013 Jun 28;8(6):e67421. doi: 10.1371/journal.pone.0067421 (PMC3696098; doi:10.1371/journal.pone.0067421)
Supplement: Table S1 — Clinical data and IDH status of 53 patients with paired primary and recurrent gliomas. (DOCX) [file pone.0067421.s002.docx]

**Supplementary Table1. Clinical data and *IDH* status of 53 patients with paired primary and recurrent gliomas**

| Patient no. | Age (year) | Sex | Tumour location | Diagnosis  (Initial tumour) | WHO grade | |  | *IDH1*/ *IDH2* status | | Progression-free survival (month) | Overall survival (month) | Patient status |
| --- | --- | --- | --- | --- | --- | --- | --- | --- | --- | --- | --- | --- |
|  |  |  |  |  | Initial tumour | Recurrent tumour |  | Initial tumour | Recurrent tumour |  |  |  |
| Group 1 |  |  |  |  |  |  |  |  |  |  |  |  |
| HS1 | 55 | M | R fronto-temporal | OAII | II | II |  | R132H | R132H | 43 | 61 | alive |
| HS2 | 28 | M | L temporal | OII | II | II |  | R132H | R132H | 42 | 59 | alive |
| HS3 | 32 | F | L frontal | AII | II | II |  | R132H | R132H | 15 | 42 | alive |
| HS4 | 67 | M | L frontal | AII | II | II |  | R132H | R132H | 16 | 20 | dead |
| PWH1 | 38 | F | L frontal | AII | II | II |  | R132H | R132H | 28.6 | 73.5 | dead |
| PWH2 | 34 | M | Corpus callosum | AII | II | II |  | R132G | R132G | 15.6 | 91.4 | dead |
| PWH3 | 46 | F | L parietal | AII | II | II |  | wt | wt | 25.6 | 46.8 | dead |
| PWH4 | 49 | M | L frontal | OII | II | II |  | R132H | R132H | 43.2 | 117.1 | alive |
| PWH5 | 43 | F | R frontal | OII | II | II |  | R132H | R132H | 50.9 | 142.1 | alive |
| PWH6 | 45 | M | R frontal | OAII | II | II |  | R132H | R132H | 25.4 | 135.2 | dead |
| PWH7 | 36 | M | L frontal | OAII | II | II |  | R132H | R132H | 34.9 | 120.6 | dead |
| PWH8 | 5 | F | L temporal | OAII | II | II |  | wt | wt | 56 | 161.6 | alive |
| HS5 | 40 | M | L temporal | AAIII | III | III |  | R132H | R132H | 54 | 77 | dead |
| HS6 | 18 | F | R cerebellum | AGIII | III | III |  | R132S | R132S | 72 | 94 | alive |
| HS7 | 60 | M | R temporal | AOIII | III | III |  | wt | wt | 16 | 32 | dead |
| HS8 | 27 | M | R temporo-parietal | AEIII | III | III |  | wt | wt | 16 | 16 | alive |
| HS9 | 62 | F | L temporal | AAIII | III | III |  | wt | wt | 6 | 14 | dead |
| HS10 | 37 | F | L frontal | AOIII | III | III |  | wt | wt | 18 | 55 | alive |
| HS11 | 57 | M | R frontal | AEIII | III | III |  | wt | wt | 39 | 53 | alive |
| PWH9 | 34 | F | R frontal | AAIII | III | III |  | wt | wt | 7.9 | 9.4 | dead |
| PWH10 | 36 | M | bifrontal | AOAIII | III | III |  | R132H | R132H | 19.6 | 37.2 | dead |
| PWH11 | 16 | M | L frontal | AOIII | III | III |  | wt | wt | 23.4 | 28.9 | dead |
| HS12 | 60 | F | L cerebellum | GBM | IV | IV |  | wt | wt | 8 | 9 | dead |
| HS13 | 63 | M | R frontal | GBM | IV | IV |  | wt | wt | 9 | 17 | dead |
| HS14 | 57 | F | L temporo-occipital | GBM | IV | IV |  | wt | wt | 8 | 11 | dead |
| HS15 | 63 | F | R fronto-parietal | GBM | IV | IV |  | wt | wt | 10 | 17 | dead |
| HS16 | 31 | M | R temporal | GBM | IV | IV |  | wt | wt | 6 | 8 | dead |
| HS17 | 28 | F | L parietal | GBM | IV | IV |  | wt | wt | 4 | 10 | dead |
| HS18 | 41 | F | R frontal | GBM | IV | IV |  | wt | wt | 31 | 43 | alive |
| HS19 | 44 | F | R occipital | GBM | IV | IV |  | wt | wt | 24 | 24 | alive |
| Group 2 |  |  |  |  |  |  |  |  |  |  |  |  |
| HS20 | 27 | M | L frontal | AII | II | III |  | R132H | R132H | 47 | 69 | alive |
| HS21 | 31 | F | T8-9 | AII | II | III |  | wt | wt | 20 | 36 | dead |
| PWH12 | 46 | F | L parietal | AII | II | III |  | R132H | R132H | 55.6 | 63.1 | dead |
| PWH13 | 38 | M | R temporal | AII | II | III |  | R132H | R132H | 12.5 | 37 | alive |
| PWH14 | 38 | M | R frontal | AII | II | III |  | R132H | R132H | 30.7 | 43.1 | dead |
| PWH15 | 48 | M | Bifrontal | OII | II | III |  | R132H | R132H | 13.9 | 47.7 | dead |
| PWH16 | 42 | F | L temporal | OII | II | III |  | R132H | R132H | 108.4 | 130 | alive |
| PWH17 | 33 | F | L frontal | OAII | II | III |  | R172K | R172K | 108.4 | 141.5 | dead |
| PWH18 | 51 | F | L frontal | OAII | II | III |  | R132H | R132H | 12.1 | 116.9 | dead |
| HS22 | 34 | F | L frontal | OAII | II | IV |  | R132H | R132H | 18 | 27 | dead |
| HS23 | 46 | M | L frontal | AII | II | IV |  | R132H | R132H | 60 | 65 | dead |
| HS24 | 47 | F | L frontal | AII | II | IV |  | R132H | R132H | 56 | 67 | dead |
| HS25 | 29 | M | L temporal | AII | II | IV |  | R132H | R132H | 70 | 81 | dead |
| PWH19 | 49 | F | L temporal | AII | II | IV |  | R132H | R132H | 65.3 | 65.6 | dead |
| PWH20 | 31 | F | L frontal | AII | II | IV |  | R132H | R132H | 68.1 | 83.2 | dead |
| PWH21 | 25 | M | R parietal | AII | II | IV |  | R132H | R132H | 25.2 | 27.4 | dead |
| PWH22 | 35 | M | R parieto-occipital | AII | II | IV |  | R132H | R132H | 33.9 | 46.9 | dead |
| HS26 | 26 | M | R temporo-occipital | AAIII | III | IV |  | wt | wt | 14 | 23 | dead |
| HS27 | 38 | M | R fronto-parietal | AAIII | III | IV |  | R132H | R132H | 22 | 35 | dead |
| HS28 | 59 | M | R temporal | AEIII | III | IV |  | wt | wt | 13 | 39 | dead |
| PWH23 | 40 | M | Frontal | AAIII | III | IV |  | R132H | R132H | 27.5 | 51.5 | dead |
| PWH24 | 27 | M | L Frontal | AAIII | III | IV |  | R132H | R132H | 58.1 | 65 | dead |
| PWH25 | 3 | M | R temporal | AAIII | III | IV |  | wt | wt | 25.4 | 33.7 | dead |

M: male; F: female; L: left; R: right; AII: diffuse astrocytoma; AAIII: anaplastic astrocytoma; OII: oligodendroglioma; AOIII: anaplastic oligodendroglioma; OAII: oligoastrocytoma; AOAIII: anaplastic oligoastrocytoma; AEIII: anaplastic ependymoma; AGIII: anaplastic ganglioglioma; GBM: glioblastoma multiforme; wt: wild type.

Group1：Recurrent tumours with same histological grade as original tumour; Group2: Recurrent tumours with progression in histological grade compared to original tumour.
